# Enhanced Pro-apoptotic Effects of Fe(II)-Modified IVIG on Human Neutrophils

**DOI:** 10.3389/fimmu.2020.00973

**Published:** 2020-05-19

**Authors:** Stefanie Graeter, Christoph Schneider, Daniëlle Verschoor, Sandro von Däniken, Frank Seibold, Nikhil Yawalkar, Peter Villiger, Jordan D. Dimitrov, David F. Smith, Richard D. Cummings, Hans-Uwe Simon, Tchavdar Vassilev, Stephan von Gunten

**Affiliations:** ^1^Institute of Pharmacology, University of Bern, Bern, Switzerland; ^2^Crohn-Colitis Zentrum, Hochhaus Lindenhofspital, Bern, Switzerland; ^3^Departement für Dermatologie, Urologie, Rheumatologie, Nephrologie, Physiologie, Inselspital Bern, University Hospital, Bern, Switzerland; ^4^Universitätsklinik für Rheumatologie, Immunologie und Allergologie, Inselspital Bern, University Hospital, Bern, Switzerland; ^5^Centre de Recherche des Cordeliers, INSERM, Sorbonne Université, USPC, Université Paris Descartes, Université Paris Diderot, Paris, France; ^6^Department of Biochemistry, Emory University School of Medicine, Atlanta, GA, United States; ^7^Department of Surgery and Harvard Medical School Center for Glycoscience, Harvard Medical School, Beth Israel Deaconess Medical Center, Boston, MA, United States; ^8^Emory Comprehensive Glycomics Core, Department of Biochemistry, Emory University School of Medicine, Atlanta, GA, United States; ^9^Department of Clinical Immunology and Allergology, Sechenov University, Moscow, Russia; ^10^Department of Immunology, Stefan Angelov Institute of Microbiology, Bulgarian Academy of Sciences, Sofia, Bulgaria; ^11^Institute of Biology and Biomedicine, N. I. Lobachevsky University, Nizhniy Novgorod, Russia

**Keywords:** neutrophil, modified IVIG, cell death, FAS, inflammation

## Abstract

Mild modification of intravenous immunoglobulin (IVIG) has been reported to result in enhanced polyspecificity and leveraged therapeutic effects in animal models of inflammation. Here, we observed that IVIG modification by ferrous ions, heme or low pH exposure, shifted the repertoires of specificities in different directions. Ferrous ions exposed Fe(II)-IVIG, but not heme or low pH exposed IVIG, showed increased pro-apoptotic effects on neutrophil granulocytes that relied on a FAS-dependent mechanism. These effects were also observed in human neutrophils primed by inflammatory mediators or rheumatoid arthritis joint fluid *in vitro*, or patient neutrophils *ex vivo* from acute Crohn's disease. These observations indicate that IVIG-mediated effects on cells can be enhanced by IVIG modification, yet specific modification conditions may be required to target specific molecular pathways and eventually to enhance the therapeutic potential.

## Introduction

Intravenous immunoglobulin (IVIG) preparations consist of polyclonal plasma-derived IgG collected from thousands of donors. As a consequence, IVIG exhibits an immense repertoire of antibodies with specificities toward a magnitude of antigens (1). Its inherent polyspecificity may provide the basis of its pluripotent anti-inflammatory effects if used as a high-dose therapy (2), whereby a broad range of different mechanisms may act in concert, also depending on the pathogenesis of the targeted disease (3–5). Accordingly, IVIG is successfully used for the treatment of a broad range of heterogenous diseases, including neutrophil-associated disorders such as Kawasaki disease, an acute febrile vasculitis syndrome (6). Neutrophils, as innate effector cells, can cause significant tissue damage and they have been linked to the pathogenesis of a number of other inflammatory disorders, such as psoriasis (7), or Crohn's disease (8). Regulation of survival is an important mechanism to control neutrophils (9, 10). In Kawasaki disease, apoptosis of circulating neutrophils is delayed, and high-dose IVIG treatment dramatically reduces blood neutrophil counts, which has been linked to IVIG-mediated apoptosis (11, 12). Indeed, we and others previously reported that IVIG has the capacity to promote death in human neutrophils by the action of specific antibodies to Siglec-9, or the classical death receptor FAS (13–16).

Different strategies have been tested to enhance the therapeutic potential of IVIG and polyclonal immunoglobulin preparations, including diversification of the repertoire of antigen specificities by mild chemical modification (3, 17). As also evidenced by crystallography, a single antibody can adopt different binding-site conformations (conformational diversity), spontaneously in absence of antigen or by an induced-fit mechanism, eventually resulting in the capacity to bind unrelated antigens (multispecificity) (18). Co-factors, such as ferrous ions, reactive oxygen species and heme have been shown to broaden the specificities of immunoglobulins (19, 20). IVIG modified by ferrous ions or heme showed superior therapeutic effects in various *in vivo* models of sepsis (21–23) or autoimmune diabetes (24).

In this article, we show using glycan array technology that immunoglobulin modification either by ferrous ions, heme or low pH results in different repertoire shifts of specificities toward carbohydrate antigens. Exposure to ferrous ions, but not heme or low pH, enhanced the proapoptotic capacity of IVIG by a FAS receptor-dependent mechanism. Neutrophils exposed to inflammatory mediators or to rheumatic arthritis joint fluid *in vitro*, or *ex vivo* from patients with active Crohn's disease, were susceptible to enhanced death by ferrous ion-exposed IVIG. However, the ferrous ion modification effect was not observed for all tested commercial IVIG preparations, which may depend on differences in immunoglobulin isolation and purification processes (25).

## Materials and Methods

### IVIG preparations

A special maltose- and albumin-free batch of Immunovenin-intact produced without incubation at acidic pH (BulBio-NCIPB Ltd., Sofia, Bulgaria) was used, as previously done (21). Further IVIG preparations included KIOVIG™ (Shire Switzerland GmbH), Intratect® (Biotest (Schweiz) AG), Endobulin S/D (Baxter, Deerfield IL, USA) and Octagam 10% (Octapharm, Lachen, Switzerland). Modification with protein-destabilizing agents was performed as previously described (19–21, 26). Briefly, for modification with pH4, native Immunovenin-intact (10 mg/ml) was incubated 5 min at room temperature in 0.1 M sodium acetate buffer (Sigma-Aldrich, St. Louis MO, USA). Subsequently, IVIG was dialyzed 3 times against phosphate buffered saline (PBS, pH 7.2); for 1 h and then 2 times for 12 h. For the treatment with heme, native Immunovenin-intact (10 mg/ml) was incubated for 30 min on ice with 10 mM heme and subsequently dialyzed for 12 h against PBS. For exposure to ferrous ions, IVIG preparations, albumin (Sigma-Aldrich) (each at 10 mg/ml) or vehicle control were incubated 1 h at 4°C with 10 mM FeSO_4_ (Sigma-Aldrich) and subsequently dialyzed for 12 h against 4 mM EDTA in PBS and then twice against PBS for 12 h. After modification and dialysis, all IVIG preparations were sterile filtered at 0.22 μm (Filter-Bio, Huberlab, Aesch, Switzerland) and concentrated using Amicon Ultra® centrifugal filters (Merck-Millipore, Darmstadt, Germany). Antibody concentrations were determined using NanoDrop™ technology.

### Cell Isolation and Cell Culturing

Neutrophils were isolated from peripheral blood drawn from healthy donors or from patients by density gradient centrifugation, as previously described (27). Written consent was obtained from all donors and the study was approved by the medical ethics committee of the canton Bern. Briefly, granulocytes and erythrocytes were separated from peripheral blood mononuclear cells (PBMCs) by density gradient using Pancoll human, density 1.077 g/mL (PAN-Biotech, Aidenbach, Germany). Lysis with erythrocyte lysis solution (150 mM NH_4_Cl, 10 mM KHCO_3_, 0.1 mM EDTA, pH 7.3) resulted in granulocyte populations containing at least 95% neutrophils. Cells were cultured at 1 × 10^6^/mL in the presence or absence of cytokines and/or antibodies for the indicated times using complete culture medium (RPMI 1640 containing 10% FCS and 200 IU/mL penicillin/100 μg/mL streptomycin; Thermo Fisher Scientific, Waltham MA, USA). Unless otherwise indicated, cells were stimulated with 20 mg/mL IVIG (133.3 μM). Cytokine stimulation occurred 25 min before the addition of IVIG. GM-CSF (25 ng/mL; Novartis Pharma GmbH, Nürnberg, Germany and Sigma-Aldrich), LPS (100 ng/mL, Sigma-Aldrich), (z)–Val-Ala-Asp (VAD)–fluoromethylketone (ZVAD-fmk, 50 μM; BD Life Sciences, Franklin Lakes NJ, USA), quinoline-Val-Asp-difluorophenoxymethylketone (Q-VD-OPh, 20 μM; MP Biomedicals, Solon OH, USA), anti-FAS/CH11 monoclonal antibody (mAb) (MBL International Corporation, Sunnyvale CA, USA) at 20 μg/ml, were used. For conditioned medium, rheumatoid arthritis joint fluids were filtered (40 μm pore) and the cellular compartments were removed by centrifugation. The resulting supernatant was added in a 25 or 50% proportion into RPMI to prime the neutrophils for 30 min before IVIG treatment. Vehicle controls of ultra-filtered, IVIG-free vehicle of native or ferrous ion treated preparations were used. Surface staining with anti-FAS ligand mAb (Biolegend, San Diego CA, USA) was performed by flow cytometric analysis (FACSVerse; BD Biosciences, San Jose CA, USA).

### Determination of Cell Death and Apoptosis

Cell death was assessed by uptake of 1 μM ethidium bromide (Invitrogen, Carlsbad CA, USA) or propidium iodide (PI; Sigma-Aldrich) and subsequent flow cytometric analysis (FACSVerse or FACSLyric; BD Biosciences) as previously described (27), and after 20 h if not stated otherwise. Redistribution of phosphatidylserine (PS) in presence of propidium iodide (Sigma-Aldrich) was assessed by flow cytometry, as previously described (27). Recombinant His6-tagged GFP-Annexin-V was a kind gift from Prof. T. Kaufmann from the University of Bern, Switzerland. In analogy to specific lysis (28), specific cell death was calculated as follows:

Specific death [%] = (experimental death [%]—spontaneous death [%]) / (100—spontaneous death [%]) × 100.

### Mitochondrial Potential

Mitochondrial potential was assessed using tetramethylrhodamine, ethyl ester (TMRE) mitochondrial membrane potential assay kit (Abcam, Cambridge, UK), according to the manufacturer's protocol. Briefly, freshly isolated human neutrophils were incubated for 5 h with or without IVIG preparations. Carbonyl cyanide-4-(trifluoromethoxy)phenylhydrazone (FCCP) at 50 μM was used as control. The staining was assessed by flow cytometry (FACSVerse; BD Biosciences).

### Preincubation of IVIG

Preincubation experiments were performed as previously described (16). Briefly, the indicated concentrations of IVIG were incubated on ice for 45 min with recombinant FAS-Fc (10 μg/mL), TNF receptor 1 (TNF-R1)-Fc (10 μg/mL), both from Enzo Life Sciences AG (Lausen, Switzerland), Siglec-9-Fc (10 μg/mL) from R&D Systems (Minneapolis MN, USA) or complete culture medium, and subsequently used in neutrophil cultures. In other experiments, indicated concentrations of IVIG were incubated in complete culture medium in the presence and absence of neutrophils (5 × 10^6^/mL) for 1 h. Neutrophils were subsequently removed by a centrifugation step at 2,000 rpm for 5 min, and freshly isolated neutrophils (1 × 10^6^/mL) were cultured in supernatant.

### Immunodot Assay

The binding of native and Fe(II)-IVIG to FAS was determined by a dot blot assay on nitrocellulose membrane (Millipore, Bedford, UK). Recombinant FAS (Enzo Life Sciences AG) in PBS was dotted at 10 μg/mL on nitrocellulose filter membranes. Dots were blocked with PBS containing 0.05% Tween 20 (Sigma-Aldrich) and 5% bovine serum albumin (BSA; Sigma-Aldrich) and incubated with IVIG preparations (20 mg/mL) overnight at 4°C. The membranes were washed with PBS containing 0.05% Tween 20. Quantification of FAS-specific IVIG antibodies was done by incubation with anti-human IgG1 (Biotin-SP-conjugated AffiniPure goat anti-human IgG + IgM, Jackson ImmunoResearch, West Grove PA, USA, 1:20'000 in PBS/T) and subsequent detection with horseradish peroxidase-conjugated streptavidin (DAKO, Glostrup, Denmark, 1:3'000 in PBS/T). Membranes were soaked in enhanced chemiluminescence detection reagent (Millipore; Burlington MA, USA) and visualized using the luminescent image analyzer LI-COR® Odyssey (Lincoln NE, USA).

### Elisa

Ninety-six well-polystyrene plates (Thermo Scientific™ Immuno non-sterile 96-well Nunc MaxiSorp™ flat-bottom) were coated for 1 h at room temperature with 2 μg/ml of Factor VIII (Kogenate FS, Bayer, Munch, Germany), with 10 μg/ml of Factor IX (LFB Biomedicaments, Les Ulis, France), Histone 3 (Sigma-Aldrich), or Myelin basic protein (Sigma-Aldrich) in PBS buffer. Plates were blocked with 0.25% Tween 20 in PBS for 90 min. After the plates were incubated 100 μg/ml of pooled IgG preparations for 2 h at room temperature. After washing with PBS containing 0.05% Tween 20, mouse anti-human IgG antibody conjugated HRP (Southern Biotech, Birmingham AL, USA) was added and incubated for 1 h at room temperature. After series of washing with PBS containing 0.05% Tween 20, immunoreactivities were revealed by adding o-phenylenediamine dihydrochloride (Sigma-Aldrich) diluted in phosphate citrate buffer pH 5. The absorbance values were read at 492 nm after stopping of the reaction with 2N HCl.

### Glycan Array Analysis

The glycan microarrays from the CFG (http://www.functionalglycomics.org/static/consortium/resources/resourcecoreh8.shtml) were prepared from amine functionalized glycan structures covalently coupled in microarrays to N-hydroxysuccinimide-derivatized microscope slides as previously described (29). The IVIG preparations were screened at 180 ug/ml for binding to glycans on CFG glycan array version 5.1 (610 different glycans), as previously described (1).

### Statistical Analysis

Statistical analysis was performed as indicated in the figure legends using GraphPad Prism versions 6.0c and 8.0.1 (GraphPad Software Inc., San Diego CA, USA). Heatmap and hierarchical clustering were performed using R (The R Foundation for Statistical Computing, Version 3.0.2).

## Results

**Figure 1:**
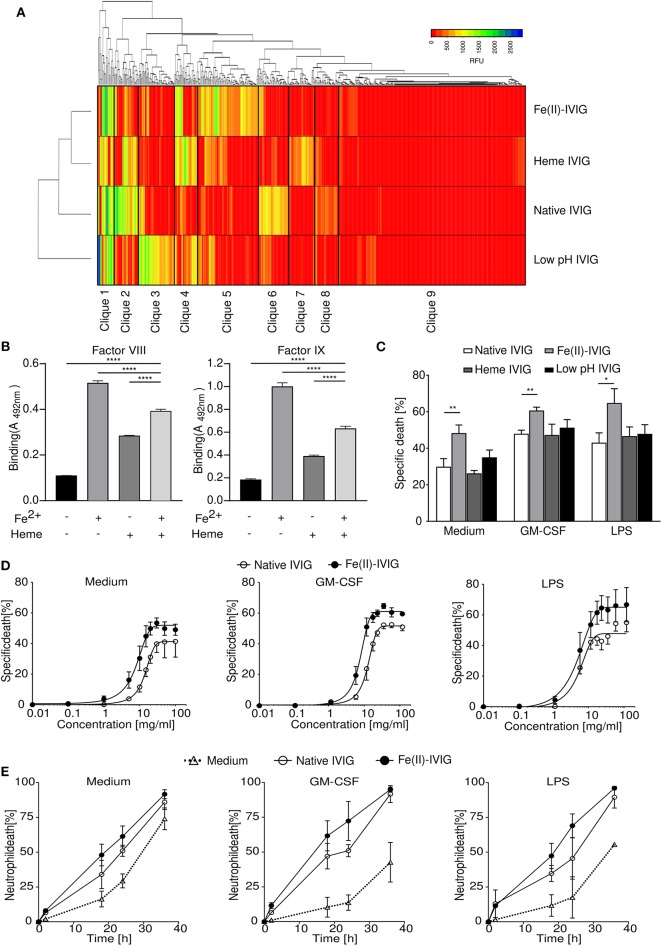
Immunoglobulin modification by ferrous ions, heme or low pH results in a diverse immunoprofile with altered capacity for regulating neutrophil survival. **(A)** Immunoprofiles of ferrous ion-exposed Fe(II)-IVIG, heme- or low pH-exposed IVIG preparations, analyzed by glycan array technology. Hierarchical clustering analysis based on reactivity levels expressed as relative fluorescence units (RFU) and as indicated in the color key. **(B)** Binding reactivity to factor VIII and IX of IVIG before and after exposure to heme and/or ferrous ions as analyzed by ELISA. **(C–E)** Neutrophil death upon treatment with 20 mg/ml native or modified IVIG (20-h cultures), analyzed by flow cytometry. **(C)** Death-promoting effects of modified IVIG preparations on neutrophils in presence or absence of GM-CSF or LPS. **(D,E)** Concentration effect curve **(D)** and time course **(E)** for cell death induction by ferrous ion-exposed Fe(II)- or native IVIG in unprimed or primed (GM-CSF, LPS) neutrophils analyzed by flow cytometric ethidium bromide exclusion assay. **(D)** Specific death in 20-h cultures calculated in comparison to untreated controls as outlined in the Materials and Methods section. **(E)** Neutrophil death upon treatment with 20 mg/ml IVIG. Two-way ANOVA, followed by Dunnett's posttest **(B)** or Tukey's posttest for comparisons among groups **(C)**. Data are representative of three **(E)**, four **(B)**, or at least five **(C,D)** experiments; mean ± SD **(B)** or SEM **(C–E)**. *P < 0.05, **P < 0.01, ****P < 0.0001. Specific death was calculated in comparison to untreated controls as outlined in the Materials and Methods section.

The modification of antibodies using protein destabilizing agents has been shown to alter their antigen recognition capacity (19, 20). To compare the impact of different protein destabilizing agents on the immunoprofiles of an IVIG preparation we used glycan array version 5.1 from the Consortium for Functional Glycomics (CFG), which permits repertoire analysis for binding to 610 glycan antigens (1, 30). A previously described early production stage batch of Immunovenin-intact (native IVIG) was screened, which was not pre-exposed to acidic pH during production and was maltose- and albumin-free (21). As revealed by hierarchical clustering analysis, exposure of this native IVIG to ferrous ions, heme or pH 4 conditions resulted in diverse immunoprofiles with distinct carbohydrate recognition patterns and divergent cliques of highly correlated reactivities (Figure 1A). Our analysis revealed that during the modification process specific reactivity of antibodies to certain antigens is lost (cliques 2 and 6) or gained (cliques 3, 5, or 7). Furthermore, combination treatment of IVIG by heme or ferrous ions differentially affected the binding of IVIG to factor VIII, factor IX, as compared to single modification (Figure 1B). Similar effects were found for IVIG binding to the antigens histone 3 and myelin basic protein (Supplementary Figure 1).

Regulation of neutrophil survival by IVIG at concentrations achieved during high-dose IVIG therapy, has been ascribed to the activities of specific functional antibodies, and IVIG-induced neutrophil death is enhanced under inflammatory conditions (13, 15, 16, 31). Given the different repertoire changes induced by the various protein destabilizing factors, we compared the effects of native IVIG with ferrous ion-, heme- or low pH-exposed preparations on neutrophil survival (Figure 1C). While all these IVIG preparations at 20 mg/ml exhibited higher neutrophil death in presence of GM-CSF or LPS, IVIG modification by ferrous ion exposure significantly enhanced the death-inducing capacity of IVIG. Neither native nor ferrous ions exposed vehicle control, EDTA controls, or albumin had an effect on neutrophil survival (Supplementary Figure 2). Among conventional commercial IVIG preparation, the enhanced death effect upon ferrous ion exposure was observed for one further preparation (IVIG #1) (Supplementary Figure 3), which could be due to differences in titers of anti-FAS antibodies among preparations (32), or repertoire shifts due to protein-modifying conditions during specific IVIG production processes (25). However, we continued our study using the early stage preparation of Immunovenin-intact, as described above.

The enhanced death response of neutrophils to ferrous ion-modified IVIG “Fe(II)-IVIG,” was already observed at concentrations below 10 mg/ml (Figure 1D), indicating an increase of both efficacy and potency. Neutrophil death upon Fe(II)-IVIG treatment was both concentration- (Figure 1D) and time-dependent (Figure 1E) under unstimulated or inflammatory conditions.

### Enhanced Pro-Apoptotic Effects of IVIG Upon Ferrous Ion Exposure

**Figure 2:**
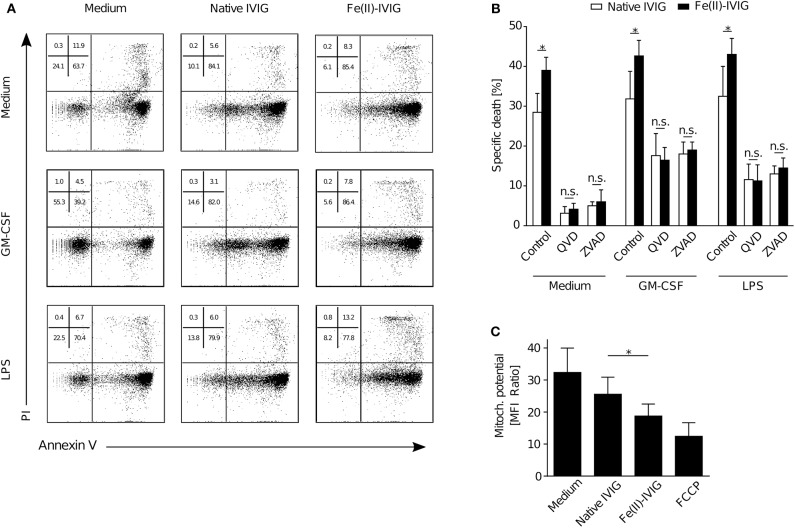
Modification of IVIG by ferrous ion-exposure enhances pro-apoptotic effects of IVIG in both unprimed and primed (GM-CSF, LPS) neutrophils. Flow cytometric analysis of neutrophils upon treatment with Fe(II)- or native IVIG. **(A)** Representative Annexin V-FITC/PI staining of 15 h-cultures of unprimed or primed (GM-CSF, LPS) neutrophils. **(B)** IVIG induced neutrophil death upon pretreatment with the pan-caspase inhibitors Q-VD-OPh (QVD) or Z-VAD-fmk (ZVAD) in 20 h-cultures of unprimed and primed (GM-CSF, LPS) cells. **(C)** Mitochondrial potential assessed by tetramethylrhodamine ethyl ester (TMRE) staining in 5 h cultures. Carbonyl cyanide-4-(trifluoromethoxy)phenylhydrazone (FCCP) was used as a positive control. Two-way ANOVA, followed by Tukey's posttest for comparisons among groups **(B)**, or paired *t* test **(C)**. Data are representative of at least 2 **(B)**, three **(A)**, or four **(C)** independent experiments (mean ± SEM in **B,C**). *P < 0.05, n.s., non-significant.

Both apoptotic and non-apoptotic forms of neutrophil death have been described, depending on the inflammatory environment (13, 15). Fe(II)-IVIG treatment was associated with apoptotic features such as increased Annexin-V staining (Figure 2A), as assessed by flow cytometry. To confirm that the enhanced cytotoxic capacity of IVIG by ferrous ion exposure is apoptotic, pharmacological inhibition experiments using the pan-caspase inhibitors Q-VD or z-VAD were performed in presence or absence of the pro-inflammatory stimuli GM-CSF or LPS (Figure 2B). In line with previous evidence (13), pan-caspase inhibition abrogated IVIG-induced death in unprimed neutrophils, but had only a partial effect on cell death in presence of GM-CSF or LPS, indicating residual non-apoptotic death under these conditions. However, upon caspase inhibition the enhanced death upon Fe(II)-IVIG treatment was no longer observed, neither in unprimed nor in GM-CSF or LPS-treated cells indicating a requirement of caspase-dependent pathways. Furthermore, the mitochondrial potential was significantly lower upon culture of neutrophils in presence of Fe(II)-IVIG (Figure 2C). Taken together, these findings indicate that the increased neutrophil death-inducing capacity of IVIG upon ferrous ion exposure depends on apoptosis.

### Enhanced Anti-FAS Activity of Fe(II)-IVIG on Neutrophils

**Figure 3:**
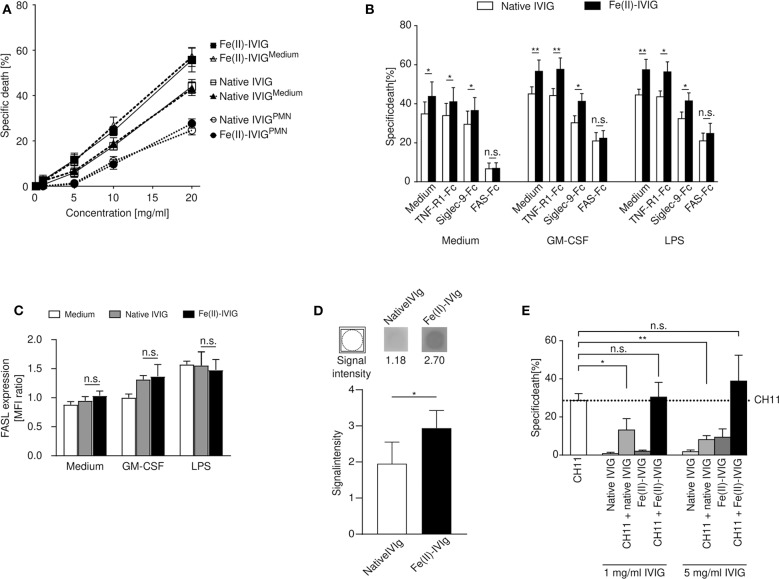
Increased cytotoxicity of ferrous ion-exposed IVIG involves FAS signaling in neutrophils. **(A)** Concentration-dependent death response of autologous neutrophils to IVIG (native or ferrous ion-exposed) following preincubation with polymorphonuclear neutrophils (PMNs) (IVIG^PMN^) or medium (IVIG^medium^). **(B)** Neutrophil death upon preincubation of native or Fe(II)-IVIG with recombinant TNF-R1-, Siglec-9- or FAS-Fc proteins in presence of absence of GM-CSF or LPS. **(C)** Surface expression of FAS ligand (FASL) on unprimed or primed (GM-CSF or LPS) neutrophils upon culture for 15 h in medium, native or Fe(II)-IVIG. **(D)** Immunoblotting indicating native and Fe(II)-IVIG reactivity to immobilized recombinant FAS-Fc protein. **(E)** Blocking effect of IVIG preincubation at two different concentrations on neutrophil death induced by an α-FAS monoclonal antibody (mAb, clone CH11). The dashed line represents the mean level of neutrophil death specifically induced by α-FAS mAb treatment. Students *t* test **(B)**, paired *t* test **(D)**, one-way ANOVA, followed by Tukey's posttest for comparisons among groups **(C)**, or Dunnett's posttest with anti-FAS as control **(E)**. Data are representative of three **(A)**, four **(D)** five **(C)**, or six **(B,E)** independent experiments (mean ± SEM). *P < 0.05, **P < 0.01, n.s., non-significant. Specific death was calculated in comparison to untreated controls as outlined in the Materials and Methods section.

Neutrophil survival is regulated by cell surface receptors, such as FAS, TNF-R1 or Siglec-9 (33, 34). Preincubation of IVIG with neutrophils has been shown to diminish the cytotoxicity of IVIG, suggesting a role of specific antibodies to surface receptors contained in IVIG (16). Pre-adsorption of both native or Fe(II)-IVIG with neutrophils aimed at reducing antibodies to neutrophil surface molecules diminished the cytotoxic capacity of both preparations (Figure 3A). Using recombinant Fc-coupled TNF receptor 1 (TNF-R1), FAS, or Siglec-9 proteins, the involvement of specific surface receptors in native or Fe(II)-IVIG induced death was investigated in blocking experiments (Figure 3B). Siglec-9-Fc blocked the death of GM-CSF- or LPS-primed, but not unprimed neutrophils, which is expected given that cross-linking of Siglec-9 has been shown to transduce cytokine-dependent neutrophil death (35, 36). However, exclusively FAS-Fc abrogated the enhanced cytotoxic activity of Fe(II)-IVIG, an effect that was observed in all cells, unprimed or GM-CSF or LPS stimulated. This suggests that FAS-dependent pathways are primarily responsible for modification-related effects, while other death-inducing stimuli might co-exist. Flow cytometric analysis did not indicate a difference of FAS ligand (FASL) surface expression between native or Fe(II)-IVIG (Figure 3C). However, immunoblotting revealed higher binding activity of Fe(II)-IVIG to FAS than native IVIG (Figure 3D and Supplementary Figure 4). The paralleled increase of FAS reactivity and FAS-dependent death by Fe(II)-IVIG indicated that the altered specificity of modified IVIG enhances the proportion of FAS-specific antibodies with agonistic properties.

Given that IVIG has been reported to contain both agonistic and blocking antibodies to FAS (16, 37), we also examined the capacity of Fe(II)-IVIG to block the pro-apoptotic effects of a FAS-specific monoclonal antibody, clone CH-11, on neutrophils (16, 36). While native IVIG at 1 and 5 mg/ml inhibited CH11-induced neutrophil death, the blocking capacity was lost in Fe(II)-IVIG at both concentrations (Figure 3E). These data suggest that enhanced FAS signaling by Fe(II)-IVIG cannot be explained exclusively by an increase of FAS-binding capacity (Figure 3D) in isolation, but also involves a reduction of antibodies with FAS-blocking activities (Figure 3E). Thus, modification of IVIG by ferrous ion exposure may reset the balance between agonistic and blocking anti-FAS antibodies.

### Effect of Fe(II)-IVIG on Neutrophils From Inflammatory Diseases *ex vivo*

**Figure 4:**
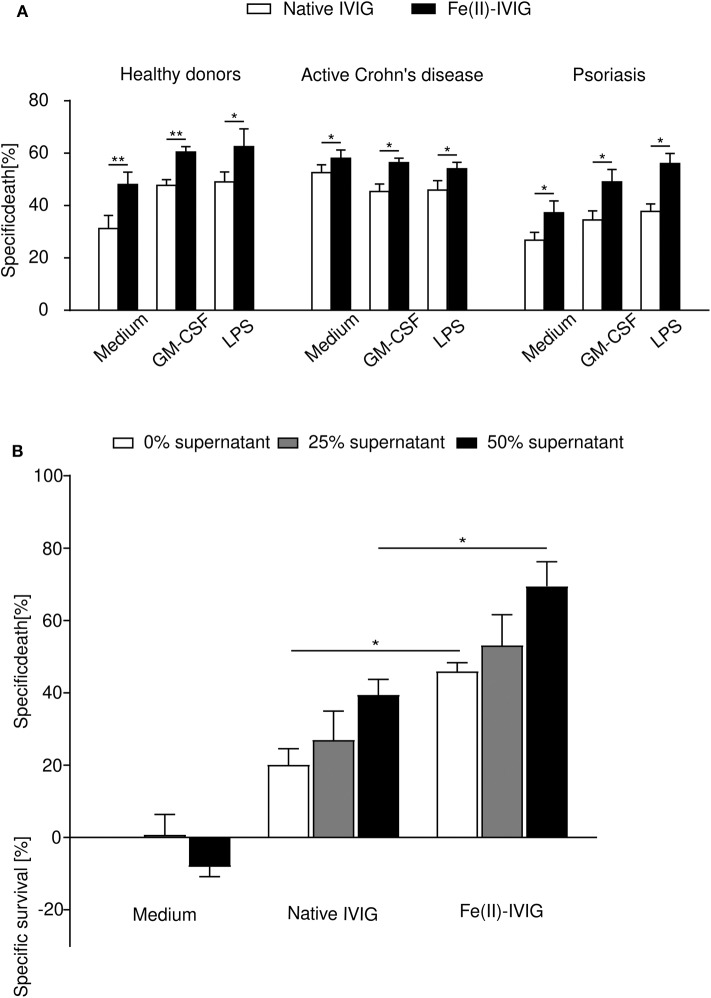
Fe(II)-modification of IVIG enhances death of neutrophils under inflammatory conditions. **(A)** Human neutrophils freshly isolated from the peripheral blood of patients with active Crohn's disease (*n* = 3), psoriasis (*n* = 5), or healthy individuals (*n* = 5) were assessed for neutrophil death (24-h cultures; flow cytometric ethidium bromide-exclusion assay) induced by native or Fe(II)-IVIG with or without prior priming with GM-CSF or LPS. **(B)** Cell death of healthy neutrophils cultured in conditioned medium with 25 or 50% cell-free rheumatoid arthritis joint fluid, in presence or absence of native or Fe(II)-IVIG, analyzed as in **(A)**. Students *t* test **(A)**, one-way ANOVA, followed by Tukey's posttest for comparisons among groups **(B)**. Data are representative of at least three **(A)** or four **(B)** independent experiments (mean ± SEM). *P < 0.05, **P < 0.01.

Neutrophils from patients with inflammatory disorders are exposed to inflammatory mediators *in vivo*, and exhibit altered survival properties when exposed to certain death-inducing stimuli (27, 35, 36). To examine the death-inducing capacity of ferrous ion-exposed IVIG on *in vivo* primed cells, death responses of neutrophils from patients with active Crohn's disease (*n* = 3) or psoriasis (*n* = 5), or healthy individuals (*n* = 5), were compared upon *ex vivo* culture and treatment with native or Fe(II)-IVIG (Figure 4A). Enhanced death responses to native IVIG were observed in neutrophils from Crohn's disease but not from psoriasis, presumably reflecting higher systemic exposure to inflammatory mediators *in vivo*. Under all conditions tested, Fe(II)-IVIG exhibited higher death responses. GM-CSF or LPS stimulation further enhanced death responses to Fe(II)-IVIG in healthy donor and psoriasis neutrophils, but not Crohn's disease neutrophils, which may be maximally stimulated *in vivo*. Conditioned medium containing 25% or 50% of cell-free joint fluid from rheumatoid arthritis (RA) patients, enhanced IVIG-induced death, whereby both the potency and efficacy of ferrous ion-exposed IVIG were higher (Figure 4B). These data suggest that IVIG modification by ferrous ion exposure may enhance the pro-apoptotic capacity of IVIG on neutrophils in inflammatory disorders.

## Discussion

It has previously been shown that modification of polyclonal IgG by protein destabilizing agents, such as ferrous ions, reactive oxygen species or heme, leads to increased immunoreactivity (19, 20). Notably, exposure of IVIG to ferrous ions resulted in the newly acquired ability to bind human cytokines, complement components and danger molecules (21). Given that array technology allows for high-throughput profiling of antibody repertoires (1, 15, 30, 38–40), here we employed glycan array technology to compare immunoprofiles upon modification of IVIG by ferrous ions, heme or low pH exposure. The various types of modification resulted in remarkable but heterogenous shifts of the repertoires of specificities, indicating that these modifying agents exerted idiosyncratic effects on the conformational diversity of a considerable proportion of antibodies contained in IVIG. Exposure to ferrous ions, but not to heme or low pH, enhanced the pro-apoptotic effects of IVIG on human neutrophils by a FAS-dependent mechanism.

Our study supports the notion that mild modification may alter the specificity repertoire of IVIG to acquire immunoregulatory characteristics. In this regard, the enhanced pro-apoptotic effects of Fe(II)-IVIG suggest that iron exposure may enhance the anti-inflammatory potency and efficacy of IVIG therapy in patients with neutrophil-predominant disorders, such as Kawasaki disease. However, future studies will be required to investigate the potential of modified IVIG in Kawasaki disease, or other inflammatory disorders such as psoriasis or Crohn's disease with pathogenetic neutrophil involvement. However, the fractionation and virus-inactivation steps in the production of IVIG would require special attention in terms of the sometimes considerable protein-modifying processes, which may affect the reactivity of the polyclonal antibodies (25), eventually depending on idiosyncratic characteristics of their conformational diversity (18). Indeed, modification by iron exposure did not enhance the pro-apoptotic activities of all commercial IVIG preparations investigated, which may be explained by the observed dependence on a distinct death pathway involving FAS (but not Siglec-9 or TNF-R1). Thus, our data support the concept that mild protein-modification can lead to enhanced anti-inflammatory effects, but that the nature of the protein-modifying agents and the pre-conditions of the IVIG fractionation process should be considered.

More than 20 years ago, the presence of anti-FAS antibodies in IVIG has been initially reported (37). Subsequent studies revealed the presence of both agonistic and blocking anti-FAS antibodies in IVIG (16, 31, 41), and the resulting effect on neutrophil survival was shown to be concentration-dependent. Such hormetic effects have recently been reported for tumor-directed antibodies (42). While we observed a net increase of FAS receptor binding activity of IVIG upon ferrous iron exposure, we observed a reduced blocking capacity of IVIG to the monoclonal anti-FAS antibody CH11, suggesting that FAS-mediated death by Fe(II)-IVIG depends on a higher ratio of agonistic vs. blocking anti-FAS antibodies.

*in vivo* studies using murine models of sepsis or autoimmune diabetes demonstrated an increased anti-inflammatory potential of IVIG modified by ferrous ions or heme (21–24). However, we previously observed that mouse neutrophils are resistant to IVIG-mediated death, suggesting that at least certain *in vivo* models may not adequately reflect IVIG effects on neutrophils, eventually due to species-differences of epitopes and molecular pathways (15). Further challenges using current models of humanized mice are imposed by small granulocyte fractions (around 3%) (43).

Our study provides conceptual evidence that mild modification influences the specificity, functional capacity, and pro-apoptotic effects of leukocyte regulatory antibodies in IVIG. We show that idiosyncratic differences exist between different protein modifying agents, which may also influence fractionation processes and clinical responses to currently used commercial IVIG preparations (25). However, our study also delineates novel directions for cell-directed therapeutic strategies in neutrophil-associated disorders through mild modification of IVIG.

## Data Availability Statement

The datasets generated for this study are available on request to the corresponding author.

## Ethics Statement

The studies involving human participants were reviewed and approved by Kantonale Ethikkommission Bern (KEK) EC approval number 110/06 and 224/01. The patients/participants provided their written informed consent to participate in this study.

## Author Contributions

SGu, TV, and H-US designed the study. SGr, CS, DV, and SD performed research, analyzed data, and prepared the figures. FS, NY, PV, TV, and JD provided material. RC and DS supervised the glycan array experiments. SGr, CS, DV, and SGu wrote the manuscript. All authors had full access to the data, helped draft the report or critically revised the draft, contributed to data interpretation, and reviewed and approved the final version of the report.

## Conflict of Interest

The authors declare that the research was conducted in the absence of any commercial or financial relationships that could be construed as a potential conflict of interest.

## Supplementary Material

Supplementary Figure 1 **(A** and **B)** Binding reactivity to histone 3 (A) and myelin basic protein **(B)** of IVIG before and after exposure to heme and/or ferrous ions as analyzed by ELISA. **(C–F)** Binding reactivity to factor VIII **(C)**, factor IX **(D)**, histone **(E)** and myelin basic protein **(F)**, of a second IVIG preparation with reported low pH treatment (company product information) as analyzed by ELISA. Data are representative of four replications (mean ± SD). ^****^*P* < 0.0001. Two-way ANOVA, followed by Dunnett's posttest.

Supplementary Figure 2 Neither vehicle with ferrous ions nor ferrous ion-exposed albumin or leftover EDTA from dialyzing has an effect on neutrophil death as assessed by flow cytometric dye exclusion assay. **(A,B)** Cell death of unprimed or primed (GM-CSF, LPS) neutrophils cultured in presence of the ferrous ion vehicle or **(A)** or Fe(II)-IVIG dialyzed with or without EDTA **(B)**. **(C)** Treatment with native and ferrous ion-exposed albumin. Students t test **(A)**, one-way ANOVA, followed by Tukey's **(B)** posttest for comparisons among groups, paired t test **(C)**. Data are representative of two **(B)**, three **(A)** or four **(C)** experiments (mean ± SEM). n.s., non-significant. Specific death was calculated in comparison to untreated controls as outlined in the Materials & Methods section.

Supplementary Figure 3 Death-promoting effects of commercial IVIG preparations (shaded bars) and corresponding Fe(II)-IVIG (filled bars) on neutrophils in presence or absence of GM-CSF or LPS. Two-way ANOVA, followed by Tukey's posttest for comparisons among groups. Data are representative of nine independent experiments (mean ± SEM). ^****^*P* < 0.0001, n.s., non-significant. Specific death was calculated in comparison to untreated controls as outlined in the Materials and Methods section.

Supplementary Figure 4 Immunoblotting indicating native and Fe(II)-IVIG reactivity to immobilized recombinant FAS-Fc protein. Representative dot blots incubated with native (upper panel) and Fe(II)-IVIG (lower panel) shown. Each blot was coated with PBS as control.
