# Supplementary figures and images for: Enhanced Pro-apoptotic Effects of Fe(II)-Modified IVIG on Human Neutrophils
Source: Front Immunol. 2020 May 19;11:973. doi: 10.3389/fimmu.2020.00973 (PMC7248553; doi:10.3389/fimmu.2020.00973)

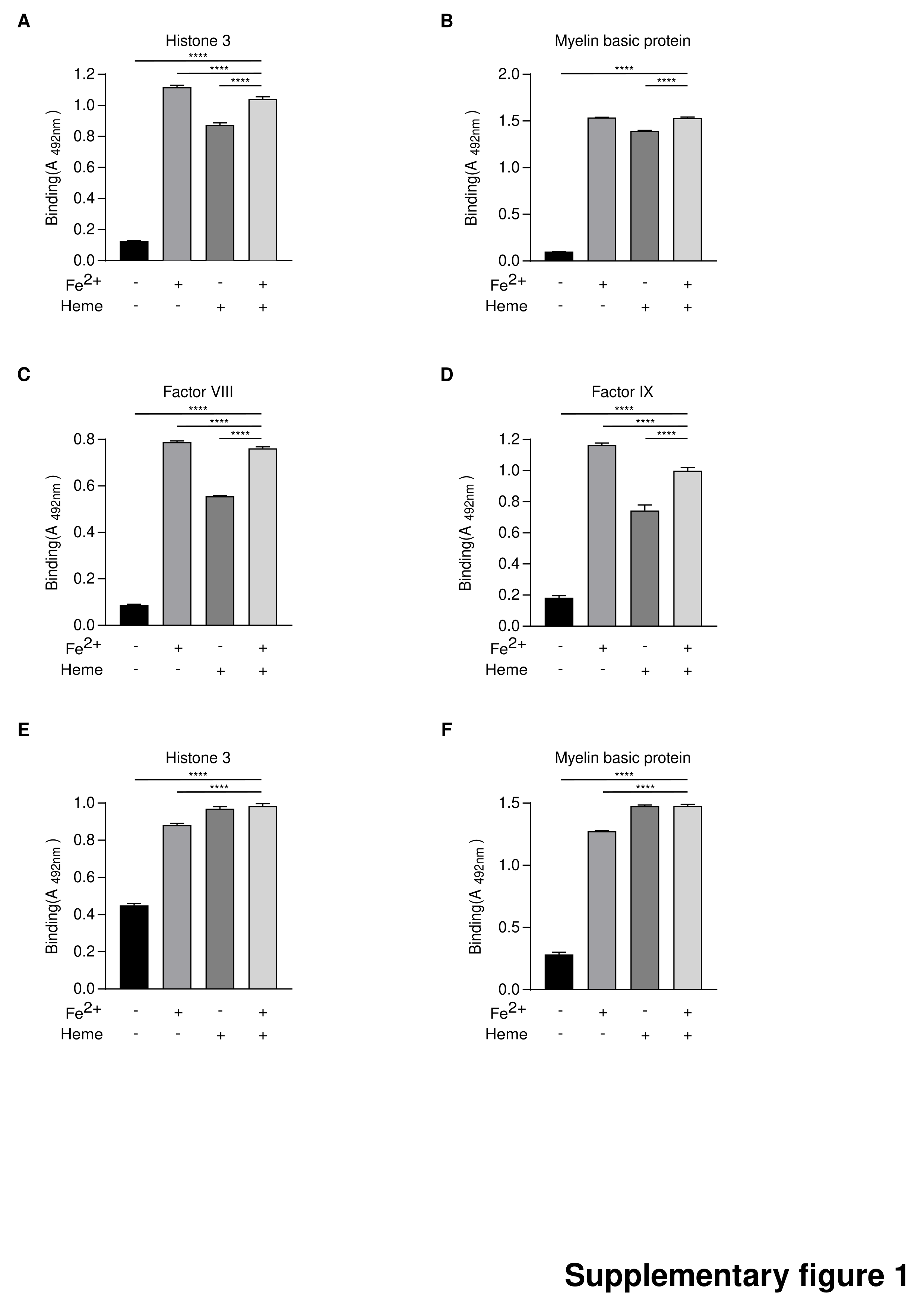

Supplement: Supplementary Figure 1 — (A and B) Binding reactivity to histone 3 (A) and myelin basic protein (B) of IVIG before and after exposure to heme and/or ferrous ions as analyzed by ELISA. (C–F) Binding reactivity to factor VIII (C), factor IX (D), histone (E) and myelin basic protein (F), of a second IVIG preparation with reported low pH treatment (company product information) as analyzed by ELISA. Data are representative of four replications (mean ± SD). ****P < 0.0001. Two-way ANOVA, followed by Dunnett's posttest. [file Image_1.tiff]

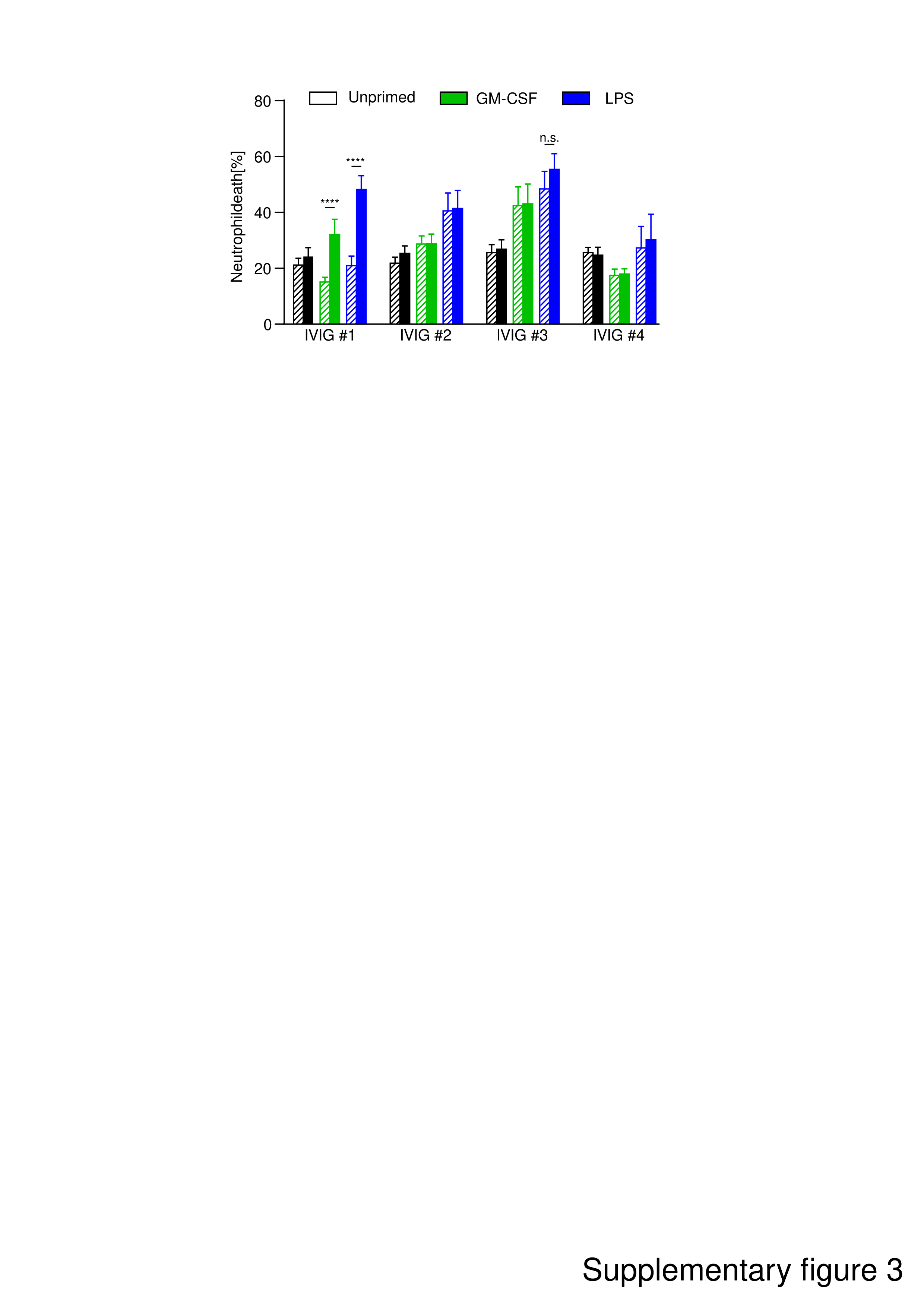

Supplement: Supplementary Figure 3 — Death-promoting effects of commercial IVIG preparations (shaded bars) and corresponding Fe(II)-IVIG (filled bars) on neutrophils in presence or absence of GM-CSF or LPS. Two-way ANOVA, followed by Tukey's posttest for comparisons among groups. Data are representative of nine independent experiments (mean ± SEM). ****P < 0.0001, n.s., non-significant. Specific death was calculated in comparison to untreated controls as outlined in the Materials and Methods section. [file Image_3.tiff]
